# Complexity Measures for EEG Microstate Sequences: Concepts and Algorithms

**DOI:** 10.1007/s10548-023-01006-2

**Published:** 2023-09-26

**Authors:** Frederic von Wegner, Milena Wiemers, Gesine Hermann, Inken Tödt, Enzo Tagliazucchi, Helmut Laufs

**Affiliations:** 1 School of Biomedical Sciences, University of New South Wales (UNSW), Wallace Wurth, Kensington, NSW 2052 Australia; 2 Department of Neurology and Clinical Neurophysiology, Lüneburg Hospital, Bögelstrasse 1, 21339 Lüneburg, Germany; 3 Department of Neurology, Christian-Albrechts University, Arnold-Heller-Strasse 3, 24105 Kiel, Germany; 4 Institute of Sexual Medicine & Forensic Psychiatry and Psychotherapy, Christian-Albrechts University, Schwanenweg 24, 24105 Kiel, Germany; 5 Department of Physics, University of Buenos Aires, 1428 Buenos Aires, Argentina

**Keywords:** Electroencephalography, EEG microstates, Complexity, Entropy, Hurst exponent, Markov models

## Abstract

EEG microstate sequence analysis quantifies properties of ongoing brain electrical activity which is known to exhibit complex dynamics across many time scales. In this report we review recent developments in quantifying microstate sequence complexity, we classify these approaches with regard to different complexity concepts, and we evaluate excess entropy as a yet unexplored quantity in microstate research. We determined the quantities entropy rate, excess entropy, Lempel–Ziv complexity (LZC), and Hurst exponents on Potts model data, a discrete statistical mechanics model with a temperature-controlled phase transition. We then applied the same techniques to EEG microstate sequences from wakefulness and non-REM sleep stages and used first-order Markov surrogate data to determine which time scales contributed to the different complexity measures. We demonstrate that entropy rate and LZC measure the Kolmogorov complexity (randomness) of microstate sequences, whereas excess entropy and Hurst exponents describe statistical complexity which attains its maximum at intermediate levels of randomness. We confirmed the equivalence of entropy rate and LZC when the LZ-76 algorithm is used, a result previously reported for neural spike train analysis (Amigó et al., Neural Comput 16:717–736, 10.1162/089976604322860677, 2004). Surrogate data analyses prove that entropy-based quantities and LZC focus on short-range temporal correlations, whereas Hurst exponents include short and long time scales. Sleep data analysis reveals that deeper sleep stages are accompanied by a decrease in Kolmogorov complexity and an increase in statistical complexity. Microstate jump sequences, where duplicate states have been removed, show higher randomness, lower statistical complexity, and no long-range correlations. Regarding the practical use of these methods, we suggest that LZC can be used as an efficient entropy rate estimator that avoids the estimation of joint entropies, whereas entropy rate estimation via joint entropies has the advantage of providing excess entropy as the second parameter of the same linear fit. We conclude that metrics of statistical complexity are a useful addition to microstate analysis and address a complexity concept that is not yet covered by existing microstate algorithms while being actively explored in other areas of brain research.

**Supplementary Information:**

The online version contains supplementary material available at 10.1007/s10548-023-01006-2.

## Introduction

EEG microstate analysis has become a widely used method to characterize spontaneous and evoked brain activity patterns (Michel and Koenig 2018). Complexity is a hallmark of integrated, large-scale brain activity and researchers have an intuition of how complexity manifests in their data, may that be neuronal spiking (Amigó et al. 2004; Szczepański et al. 2004), local field potentials (Abásolo et al. 2014), functional neuroimaging (Xin et al. 2021; Nezafati et al. 2020; Hancock et al. 2022), electroencephalography (EEG) (Casali et al. 2013), or magnetencephalography (MEG) (Fernández et al. 2011).

Yet, there is no unique theoretical notion of what complexity is or how it should be measured, and the fact that complexity is a multifaceted concept is reflected in the existence of a large number of complexity definitions in the literature (Shalizi 2006). Definitions of complexity have emerged in different scientific disciplines, and some concepts have re-emerged under different names which makes it challenging to maintain an overview of this research area (Prokopenko et al. 2008; Ay et al. 2011; Crutchfield and Feldman 2003). The specific aims of this article will be stated after a brief review of complexity concepts in general, and those used to characterize EEG microstate sequences so far. We will highlight links between these measures and to areas other than microstate research.

### Concepts of Complexity

There are at least two basic flavours of complexity, each founded on different intuitions about what represents complexity.

One concept is known as algorithmic or Kolmogorov complexity (Alekseev and Yakobson 1981) and initially defined complexity as the length of the shortest program that is able to reproduce the input data. This measure increases monotonically with the amount of ‘randomness’ in the data. Intuitively, the shortest algorithm reproducing a random sequence is a command that prints exactly that sequence and the length of that algorithm would be approximately the same as the length of the dataset. In the microstate context, this would correspond to a sequence of independent samples from the set of microstate labels, e.g., from $\{A, B, C, D\}$, possibly weighted by their relative occurrence. At the non-random end of the spectrum, a sequence that consists of a single repeated label, e.g., $AAA\ldots$, is reproduced by a very short instruction such as ’print A, *n* times’. As will be explained further below, there are more practical approaches to measure Kolmogorov complexity than trying to find the actual program, namely entropy rate and Lempel–Ziv complexity.

A separate family of complexity measures was developed because many researchers are uncomfortable with the concept of complexity being essentially the same as randomness. Following the Kolmogorov complexity concept, randomly connected neurons are more complex than a real brain, and electrical white noise is more complex than actual brain electrical activity. The ’statistical complexity’ concept, on the other hand, asks how difficult it is to obtain a statistical model of the data and aims to construct bell-shaped complexity measures when plotted against randomness (Huberman and Hogg 1986; Lindgren and Nordahl 1988). Both complexity concepts agree in that extremely ordered systems should be assigned a low complexity, as they are non-random and a compact model can often be formulated. An example of a highly ordered spatial system is a crystal structure, as is a sine wave in the world of time series. Where the two complexity concepts differ is in their assessment of highly random systems. These have high Kolmogorov complexity but their statistical complexity is low because a simple assumption (statistical independence) can already provide a good statistical model of the data (Grassberger 1986; Crutchfield 1994). A similar line of reasoning led to the concept of Tononi–Sporns–Edelman complexity which has attained attention as a theory of consciousness and brain function in general (Tononi et al. 1994).

**Fig. 1:**
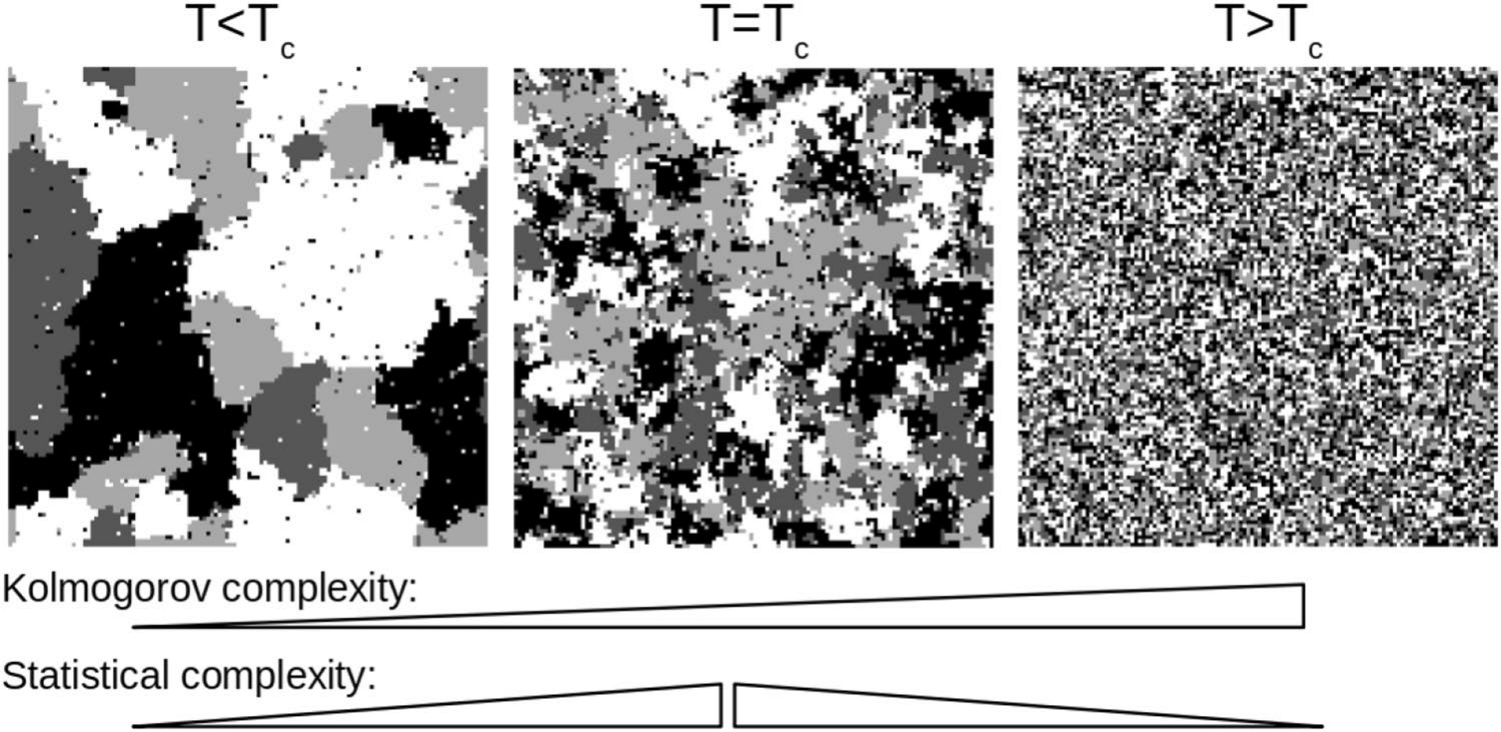
Two complexity concepts illustrated with snapshots of a 2D Potts lattice model ($Q=4$, $128 \times 128$ nodes) at different temperatures *T*. Left: the Potts model at low temperature ($0.8 \times T_c$, $T_c$ is the critical temperature) has low Kolmogorov complexity and low statistical complexity. Center: The most extensive spatial and temporal correlations occur close to the phase transition ($T=T_c$), related to intermediate Kolmogorov complexity and maximum statistical complexity. Right: Above the critical temperature ($3.0 \times T_c$), spatial features are apparently random (low order) and result in large Kolmogorov complexity (randomness) and low statistical complexity

In Fig. 1 these concepts are illustrated with snapshots of the Potts model that will be formalized further below. The model shown attains one of four discrete states on each node of a regular square lattice and the dynamics are controlled by a parameter that corresponds to thermodynamic temperature. From left to right, with increasing temperature, the spatial patterns become increasingly random and disordered. This reflects Kolmogorov complexity, which increases monotonically with temperature. Statistical complexity, however, has its peak around the critical temperature of the model ($T=T_c$) where a phase transition occurs. Around $T_c$, the most extensive spatial and temporal correlations occur and statistical forecasting of the model states is most challenging.

The two complexity concepts discussed can be quantified by a range of metrics that require some disambiguation of the historical terminology. Kolmogorov complexity can be estimated as the randomness of a signal after all correlations have been taken into account (irreducible randomness, Prokopenko et al. 2008). This is captured by the entropy rate, which is derived from joint entropy (or block entropy) estimates of signal subsequences of different lengths, or via Lempel–Ziv complexity which measures this type of complexity by compressing the signal on the basis of repeated patterns (Lempel and Ziv 1976). The more a signal can be compressed, the lower its algorithmic (Kolmogorov) complexity. Entropy rate is also known as Kolmogorov–Sinai entropy in the field of dynamical systems and chaos theory (Shalizi 2006).

Statistical complexity can be assessed by different measures. One of these measures was called statistical complexity in Crutchfield and Young (1989), but had already appeared as true measure complexity in Grassberger (1986). This quantity is based on a graph model of the underlying process that needs to be reconstructed from empirical data. As model reconstruction is not trivial, approximations can be studied instead (Grassberger 1986; Prokopenko et al. 2008). A lower bound to the true measure complexity was named effective measure entropy in Grassberger (1986) and excess entropy in Crutchfield and Feldman (1997). We will use the latter term as it is used in the recent literature and expresses Grassberger’s initial observation that finite estimates of the entropy rate converge slowly towards the asymptotic value. Excess entropy can be expressed as the surplus of entropy across all finite entropy rate estimates (Grassberger 1986; Crutchfield and Feldman 2003). The same quantity appeared as predictive information in Bialek et al. (2001) and the name refers to the interpretation that statistical complexity measures the predictability of a time series. This idea is also expressed by its information-theoretic definition as the shared information between the past and the future of a signal, relative to an arbitrary observation time point. The concept of statistical complexity has therefore also been named forecasting complexity (Zambella and Grassberger 1988).

### Complexity and Microstate Research

In the area of EEG microstate research, entropy rate, Lempel–Ziv complexity (LZC), and Hurst exponents have been explored as complexity measures in recent years. Hurst exponents were first used for microstate analysis by Van de Ville et al. (2010). Although the aim of the authors was to address self-similarity and fractality rather than explicitly measuring complexity, the relationship between Hurst exponents and complex system properties is present throughout the article. We evaluated this approach in relation to Markov models of microstate sequences in von Wegner et al. (2016) and applied it to cognitive load assessment in Jia et al. (2021).

Entropy rate estimation for microstate sequence analysis was introduced in von Wegner et al. (2018a), and we evaluated its changes during different types of cognitive effort in Jia et al. (2021), and for NREM sleep stages in Wiemers et al. (2023). We interpreted entropy rate in terms of sequence predictability in von Wegner et al. (2018a) and Jia et al. (2021), and as a complexity measure in Wiemers et al.﻿ (2023).

Next, Lempel–Ziv complexity analysis of microstate sequences was introduced in Tait et al. (2020), where a loss of complexity in the EEG of Alzheimer disease patients was demonstrated by applying a quantity called Omega complexity to the raw EEG signal (Wackermann 1996) and LZC to microstate sequences. A more recent variant of the Lempel–Ziv algorithm was used subsequently in Artoni et al. (2022) where concentration-dependent effects of propofol on microstate LZC were investigated. An intermediate approach can be found in Irisawa et al. (2006), where EEG topographies were classified with Omega complexity, and the results were compared to microstate duration, however, complexity analysis was not applied to microstate sequences.

### Aims and Outline

The specific aims of this article are (1) to evaluate an explicit measure of statistical complexity (excess entropy) on microstate data, as the existing microstate complexity studies have focused on Kolmogorov complexity, (2) to test the theoretical equivalence between entropy rate and LZC that is valid for stationary stochastic processes (Ziv 1978), (3) to compare the aforementioned measures to Hurst exponents, and (4) to test the influence of first-order Markovian correlations on these complexity measures.

As the ground truth about brain states is unknown in empirical data, we first evaluate the selected metrics (entropy rate, excess entropy, Lempel–Ziv Complexity, and Hurst exponents) on a well-understood numerical model from statistical physics, the discrete Potts model (Wu 1982).

The Potts model offers some advantages in this context. First, the model produces time series over a discrete state space with an arbitrary number of states, similar to EEG microstate sequences. The number of states is often denoted *Q* for the Potts model, and *K* in microstate research, related to the initial K-means clustering of EEG data. The second advantage of the Potts model is that there is a single control parameter (temperature) that controls the appearance of a phase transition. The common elements between the Potts model and EEG microstates are (a) entropy, which is closely linked to temperature in statistical physics, but also has an interpretation in terms of time series predictability, and (b) phase transitions have been discussed as an important feature of resting-state brain activity and are often quantified by complexity metrics such the Hurst exponent, for example in EEG research (Linkenkaer-Hansen et al. 2001; Kantelhardt et al. 2015; von Wegner et al. 2018b), EEG microstate research (Van de Ville et al. 2010; Jia et al. 2021), and fMRI studies (Bullmore et al. 2009; Tagliazucchi et al. 2013).

In the second part of the results section, we evaluate the four metrics on EEG microstate sequences in wakefulness and non-REM (NREM) sleep, a dataset we have previously analyzed with other microstate analysis tools (Brodbeck et al. 2012; Wiemers et al. 2023). We analyze full microstate sequences as well as reduced sequences from which all duplicate labels have been removed (jump sequences). In an attempt to identify which time series features the different complexity metrics actually ’see’, we use first-order Markov surrogate data to represent exactly that amount of information that is captured by the transition probability matrix, an approach that is often used to report microstate data (Lehmann et al. 2005).

## Methods

### Computational Model

In microstate research, K-means clustering is commonly performed for $K=4$ or $K=5$ clusters. Hence, we implemented the Potts model with *Q* states for $Q=4,\,5$ as reviewed in Wu (1982) on a two-dimensional (2D) discrete lattice geometry. Other topologies could be employed but the 2D model is well studied and the critical temperatures are known analytically (Wu 1982; Brown et al. 2022). Two different energy (Hamilton) functions have been presented for the Potts model, the standard model and the vector (clock) model. We chose the standard model for which the type of phase transition is known (Wu 1982).

The Potts model uses discrete variables that can be visualized as 2D unit vectors (spins), uniformly distributed around the complex unit circle. Their energy difference is defined by the phase difference. Formally, the spin values are given by $S = \{\exp \left( 2 \pi \textrm{i} q/Q \right) ,\, q=0,\ldots ,Q-1 \}$ with phase $2\pi q/Q$. For our purpose, it is sufficient to store the integer values $q=0,\ldots ,Q-1$ as the discrete model states. In the standard Potts model, a lattice site (*k*, *l*) with phase $\phi _{kl}$ has energy:

1 $$\begin{aligned} E_{kl} = - \sum _{m,n \in N_{kl}} J \delta (\phi _{kl},\phi _{mn}). \end{aligned}$$

using the Kronecker delta function ($\delta$) and nearest-neighbour coupling, i.e. the neighbours of spin $\phi _{kl}$ at lattice site (*k*, *l*) are $N_{kl} = \{(k-1,l), (k+1,l), (k,l-1), (k,l+1)\}$. We exclusively considered ferromagnetic coupling ($J = +1$) which favours neighbouring spins to align, as the lowest energies are produced when their phase values are identical. In a neuronal context, this can be interpreted as neuronal ensembles which tend to align the phase of their voltage oscillations (Breakspear et al. 2010).

We simulated the Potts model on a square lattice of $25 \times 25$ nodes. Model data were generated by Monte–Carlo simulation with Metropolis sampling, i.e., a randomly chosen $q \rightarrow q'$ transition was accepted with probability $p = \min (1, \exp \left( -\frac{\Delta E}{T}\right) )$ which depended on the energy difference $\Delta E$ before and after the proposed transition. In words, transitions that reduced the lattice energy were always accepted whereas transitions increasing the total system energy were only accepted if they could jump across the energy barrier $\Delta E$, which was tested stochastically by comparison with a uniformly distributed pseudo-random number. The critical temperature of the two-dimensional Potts model is $T_c = \left( \log (1+\sqrt{Q}) \right) ^{-1}$ (Brown et al. 2022).

Individual simulations were run for $t=30000$ iterations, preceded by a warm-up of 2500 iterations to allow relaxation from the initial random state. We simulated the system across a range of temperatures which will be written relative to the critical temperature $T_c$. We used relative temperatures $T/T_c$ of 0.2, 0.4, 0.6, 0.8, 0.9, 1.0, 1.1, 1.2, 1.4, 1.6, 1.8, 2.0, 2.2, 2.4, 2.6, 2.8, 3.0. For each temperature, we ran the model 50 times and selected a subset of $n=25$ random lattice nodes for complexity analysis. We chose a length of 30,000 samples to match the length of our EEG segments (2 min of EEG acquired at 250 Hz).

The results for $Q=4$ are presented in the main text, those for $Q=5$ in the supplementary data.

### Experimental Data and EEG Pre-Processing

We analyzed EEG recordings from n = 19 healthy subjects. The EEG dataset is a subset of the dataset analyzed in Brodbeck et al. (2012) and Wiemers et al. (2023) and only includes those subjects for whom sleep stage N3 data was available. Briefly, EEG data from simultaneous EEG-fMRI recordings were corrected for scanner and cardioballistic artefacts and downsampled to 250 Hz as described in Brodbeck et al. (2012). Sleep stages were scored manually, according to international standards. The data were band-pass filtered to 1–30 Hz with a digital Butterworth filter of order six.

### Microstate Algorithm

Subject-wise microstates were computed with the modified K-means algorithm (Pascual-Marqui et al. 1995) implemented in Python 3 (von Wegner and Laufs 2018). Group-wise microstate maps were computed for each sleep stage with a full permutation procedure over the subject-wise maps (Koenig et al. 1999), repeated 20 times with random initial conditions. Group maps were defined as the result that maximized the global explained variance across the subjects. Microstate sequences were obtained by competitive back-fitting at each time step and ignoring map polarity. No further smoothing methods were applied.

Following two different approaches found in the literature, we evaluated two types of microstate sequences, (a) full sequences as obtained from back-fitting, and (b) sequences without duplicate labels, transforming a sequence like ACCCAADBBA into ACADBA, for example. The latter approach ignores microstate duration and is rooted in the idea that the transition between non-identical brain states, as measured by EEG microstates, conveys essential information about brain activity (Michel and Koenig 2018). The latter approach is also commonly used in Markov chain analysis where these sequences are called jump sequences, a name that will also be used here (Gillespie 1992). Microstate maps and further properties of the EEG dataset used here are detailed in Wiemers et al. (2023).

### Surrogate Data

Surrogate data for the Potts model and EEG microstate sequences were synthesized as first-order Markov processes, based on the empirical transition probability matrix of each time series as explained in von Wegner et al. (2016) and von Wegner and Laufs (2018). The Markov structure of microstate sequences was quantified with partial autoinformation coefficients as described in von Wegner (2018).

### Complexity Metrics

#### Entropy Rate and Excess Entropy

**Fig. 2:**
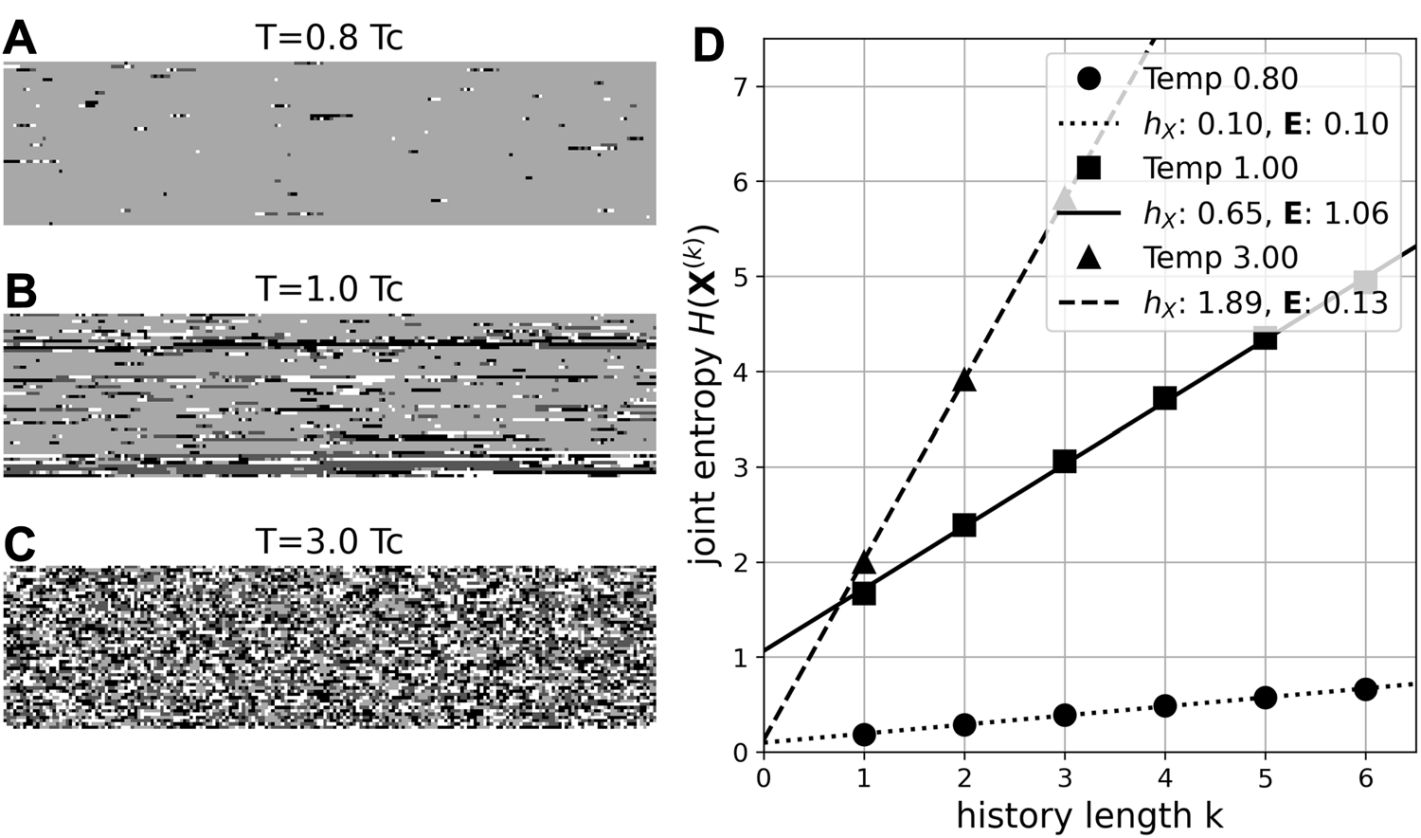
Entropy rate and excess entropy estimation. On the left (**A**–**C**) time series at random lattice nodes of the Potts model ($Q=4$) are illustrated as raster images, grey values correspond to the four Potts model states. Time runs from left to right, starting at the top row. Time series are shown at a sub-critical temperature (**A** 0.8 $T_c$), close to criticality (**B** $T \sim T_c$), and at a high temperatures (**C** 3.0 $T_c$). **D** Kolmogorov complexity and statistical complexity of the time series shown in **A**–**C** as measured by the slope (entropy rate, $h_X$) and y-axis intercept (excess entropy, ${\textbf{E}}$) of the linear fit to the joint entropies $H\left( {\textbf{X}}^{(k)}\right)$, respectively

Entropy rate and excess entropy were computed as described in von Wegner (2018) and published in our 2017 Python microstate package (github repository) (von Wegner and Laufs 2018). Briefly, the frequency distribution of microstate sequence blocks (‘microstate words’) ${\textbf{X}}_{n}^{(k)}=\left( X_{n}, \ldots , X_{n+k-1} \right)$ for each block length $k=1,\ldots ,6$ was estimated from the data, and the joint entropy $H\left( {\textbf{X}}^{(k)} \right)$ of each distribution $P\left( {\textbf{X}}^{(k)} \right)$ was computed. The parameters entropy rate ($h_X$) and excess entropy (${\textbf{E}}$) were obtained as the slope and y-axis intercept of a linear fit of $H\left( {\textbf{X}}^{(k)} \right)$ vs. *k*, respectively. This approach is visualized in Fig. 2 for three different situations of the Potts model. This estimate of the entropy rate $h_X$ is based on the following definition in terms of infinitely long observations:

2 $$\begin{aligned} h_X&= \lim _{n \rightarrow \infty } \frac{1}{n} H({\textbf{X}}_{n}^{(k)}), \end{aligned}$$

which, for stationary stochastic processes, is equivalent to the conditional entropy form:

3 $$\begin{aligned} h'_X = \lim _{n \rightarrow \infty } H(X_{n+1} \vert {\textbf{X}}_{n}^{(k)}). \end{aligned}$$

The second form (3) can be read in terms of time series predictability; $h_X$ expresses the uncertainty (entropy) in predicting the next state of the sequence ($X_{n+1}$) when the last *k* states (${\textbf{X}}_{n}^{(k)}$) are known.

In a similar approach, excess entropy can be expressed as the mutual information between the past ${\textbf{X}}_{\textrm{past}}=\left( \ldots ,X_{n-1},X_{n} \right)$ and the future ${\textbf{X}}_{\textrm{future}}=\left( X_{n+1},X_{n+2},\ldots \right)$ of the process:

4 $$\begin{aligned} {\textbf{E}} = I({\textbf{X}}_{\textrm{future}}; {\textbf{X}}_{\textrm{past}}). \end{aligned}$$

The concept can be made more intuitive by re-writing mutual information between the random variables *X*, *Y* as $I(X;Y)=H(X)-H(X \vert Y)$, i.e., the reduction in uncertainty about *X* by knowing *Y*. In this sense, excess entropy encodes to what extent predictions about the future improve, or entropy decreases, by including knowledge about the past of the process:

5 $$\begin{aligned} {\textbf{E}}&= H({\textbf{X}}_{\textrm{future}}) - H({\textbf{X}}_{\textrm{future}} \vert {\textbf{X}}_{\textrm{past}}). \end{aligned}$$

#### Lempel–Ziv Complexity (LZC)

The 1976 implementation of the Lempel–Ziv algorithm (LZ-76, Lempel and Ziv 1976) was written and compiled in Cython 0.29.3 to produce faster Python 3.6.9 code. Our implementation is identical to the algorithm used in Tait et al. (2020) which is publicly available.

#### Hurst Exponents

Hurst exponents were calculated by detrended fluctuation analysis (DFA) as described in Van de Ville et al. (2010) and von Wegner et al. (2016), using 50 logarithmically spaced time scales over the range of 50–2500 samples (200 ms—10 s for EEG data). Hurst exponents were determined as the slope parameter of the linear fit to the detrended fluctuation function. DFA was applied to random walks that were constructed by partitioning the discrete variables (Potts model states, EEG microstate classes) into two subsets, and substituting these categorical discrete variables with the values $\pm 1$, respectively (Van de Ville et al. 2010). There are three different (2, 2)-partitions for datasets with four states (Potts model for $Q=4$, EEG microstates) and ten (2, 3)-partitions for five states (Potts model for $Q=5$). For each time series, Hurst exponents were computed for each partition and the average across all partitions was used for statistical analysis.

#### Convergence

To analyze numerical differences between the entropy rate estimator $h_X$ and Lempel–Ziv complexity values for different sequence lengths, we quantified their convergence rate towards theoretically expected entropy rates using Markovian test sequences. Details of this procedure are explained in the supplementary material.

#### Code Availability

Sample Potts model data and analysis scripts to reproduce a simplified version of Fig. 3 is available online (github repository).

## Results

### Potts Model

**Fig. 3:**
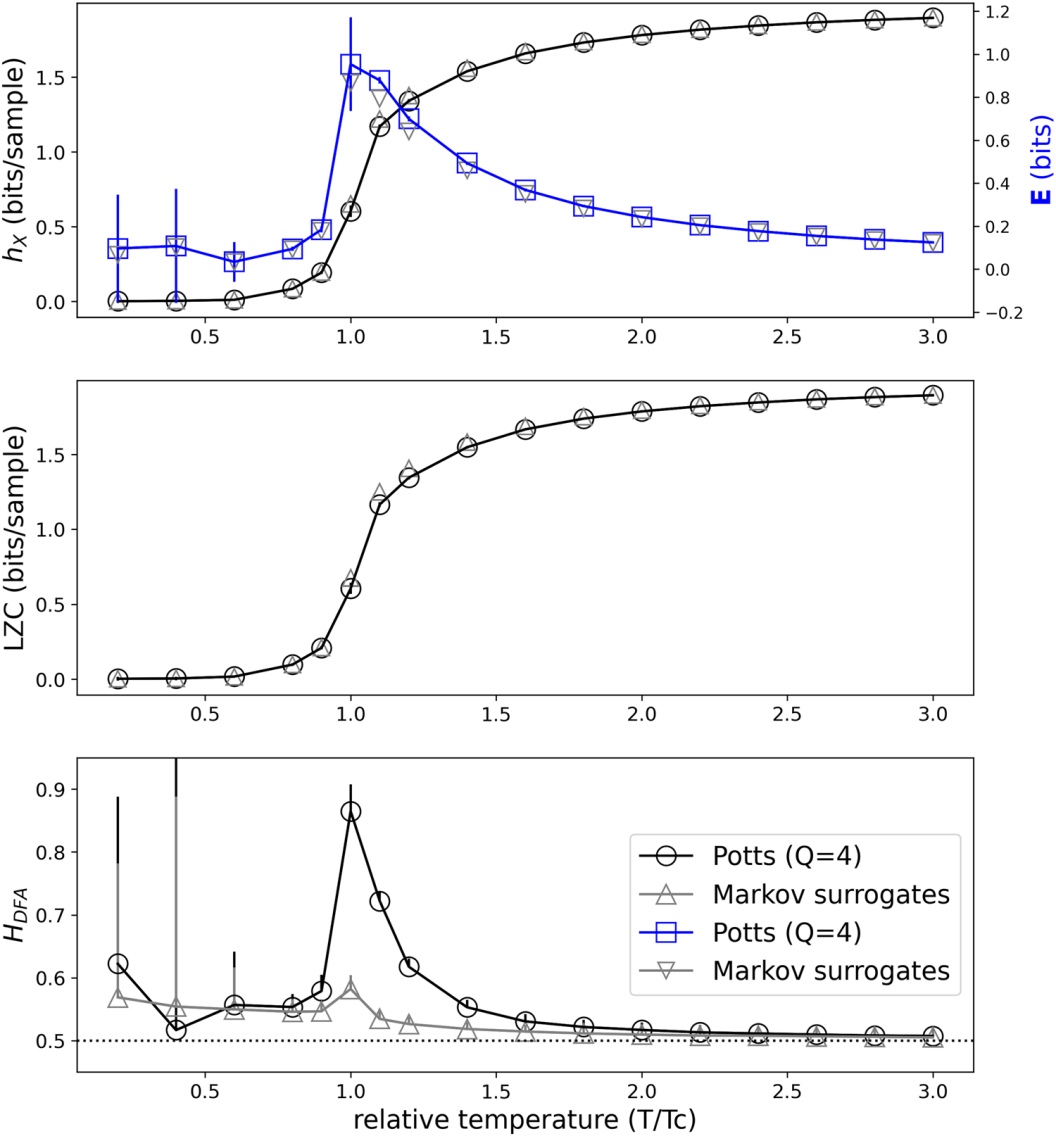
Complexity metrics for the Potts model ($Q=4$, black or blue lines/circles) and first-order Markov surrogates (grey triangles) plotted against relative temperature $T/T_c$ (critical temperature $T_c$). Error bars in all panels represent standard deviations. Error bars are omitted for surrogate statistics to simplify the visual presentation. Top: Entropy rate ($h_X$, black) and excess entropy (${\textbf{E}}$, blue) from joint entropy estimates. Center: Lempel–Ziv complexity (LZC, black) computed with the LZ-76 algorithm. Bottom: Hurst exponents ($H_{\textrm{DFA}}$) measured by detrended fluctuation analysis (DFA). Due to large variability at low temperatures, only the upper half of error bars are shown. The legend applies to all three panels

#### Entropy Rate and Excess Entropy

Results for the Potts model ($Q=4$) are shown in Fig. 3. The top panel shows entropy rate (black) and excess entropy (blue) plotted against relative temperature ($T/T_c$). The critical temperature is defined by $T/T_c=1$ on the x-axis. With increasing temperature the entropy rate rises sigmoidally and the steepest slope occurs at the critical point. At low temperatures ($T/T_c=0.2$), the time courses at most lattice sites are constant, or have very few state changes. As there is no randomness in the data, there is no prediction uncertainty and the entropy rate is close to zero. The maximum entropy rate for a *Q*-state process is $\log _2(Q)$ bits/sample, i.e., 2 bits/sample for $Q=4$. The Potts model tends towards the maximum entropy rate value, indicating that high temperature dynamics are largely random. Excess entropy peaks at the critical point and decays to lower, but non-zero values, away from the critical temperature ($T<T_c$ and $T>T_c$). Although asymmetric around the critical temperature, the shape of the excess entropy curve reflects the concept of statistical complexity whereas entropy rate reflects the Kolmogorov complexity concept. Above the critical temperature, the variability of both metrics is so small that the error bars (standard deviation) are not visible. For both metrics, the Markov surrogate datasets (grey triangles) show only minor deviations from the actual model data results. Error bars for surrogate data are equally small but are omitted from the graph to improve visibility. Analogous results were obtained for the five-state Potts model ($Q=5$) and are presented in the supplementary data, Fig. S1.

#### Lempel–Ziv Complexity (LZC)

LZC analysis of Potts model data is shown in the center panel of Fig. 3. LZC values, when scaled to bits/sample, are numerically very close to entropy rate estimates. Again, the steepest increase is found at the critical temperature and first-order Markov surrogates (grey triangles) are almost indistinguishable from Potts model data. Minor deviations are observed around the critical temperature ($T/T_c=1.0,1.1,1.2$) where surrogate data LZC is slightly larger than model data complexity. Error bars (standard deviation) are hardly visible due to extremely low LZC variability. Variability across the 1250 surrogate time series was also very low, and error bars are omitted. Results for $Q=5$ are shown in Fig. S1.

#### Hurst Exponents

Hurst exponents computed by DFA are shown in Fig. 3 (bottom panel). For the Potts models, Hurst exponents peak at the critical temperature and decay towards $H=0.5$ at high temperatures, the theoretical value for random processes. Hurst exponent variability in the low temperature range was large and only the upper half of the error bars is shown. At low temperatures, many lattice sites experienced very few state changes which appear as large jumps in the random walk embedding. These jumps appear as significant fluctuations at long time scales, leading to steep fluctuation functions and large Hurst exponents. Results for $Q=5$ are shown in Fig. S1.

### EEG Microstate Sequences in Wake and Sleep

**Fig. 4:**
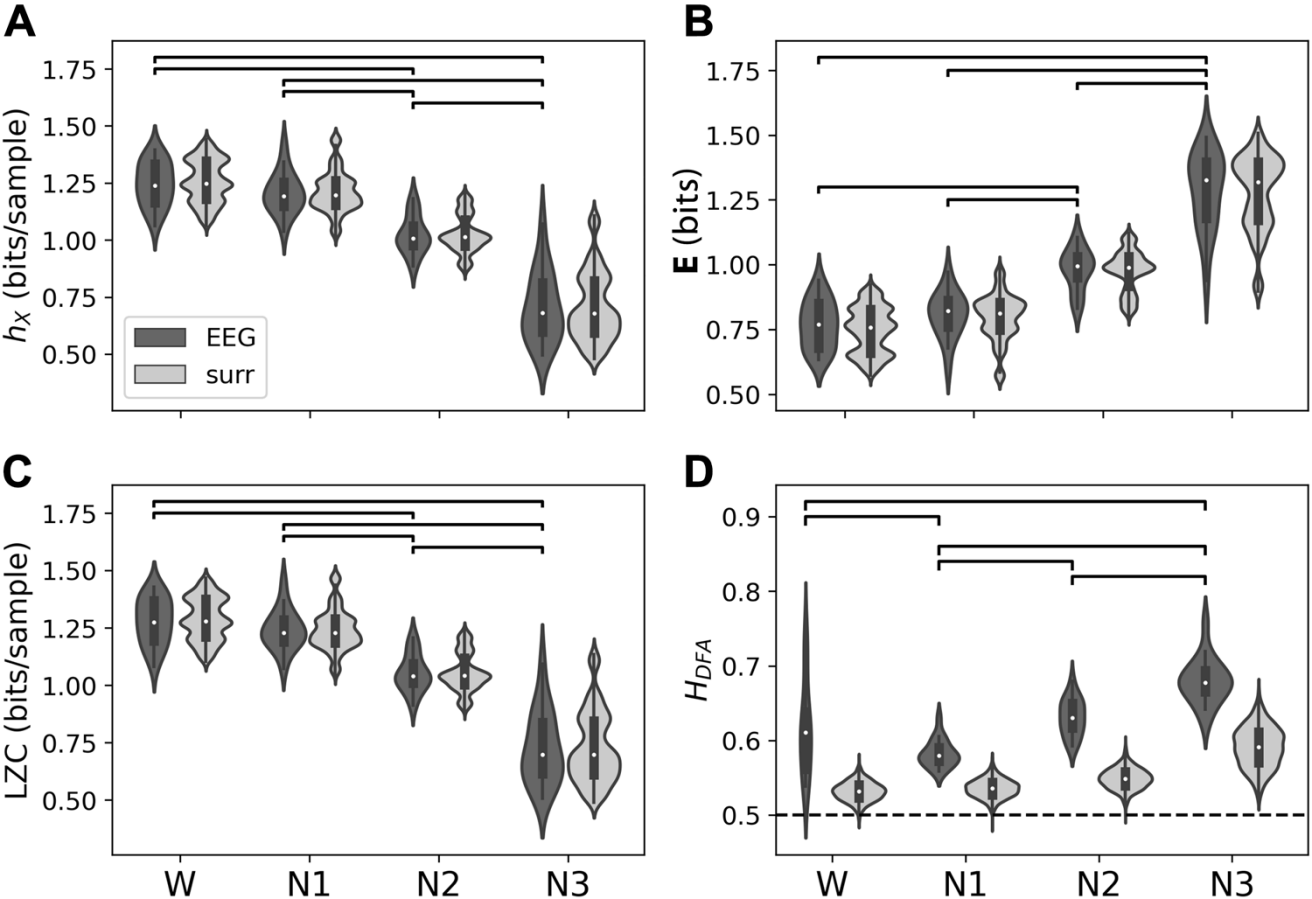
Complexity metrics for EEG microstate sequences ($K=4$) in wakefulness and NREM sleep. Results for EEG microstate sequences are shown in dark grey (label ‘EEG’), Markov process surrogates in light grey (label ‘surr’). **A** Entropy rate ($h_X$), **B** excess entropy (${\textbf{E}}$), **C** Lempel–Ziv complexity (LZC), **D** Hurst exponents from DFA ($H_{\textrm{DFA}}$). Differences between microstate sequence results were tested with one-way ANOVA (significance level $\alpha =0.05$), and post-hoc Tukey tests. Significant pairwise differences ($p_{\textrm{FWE}}<0.05$) are indicated by brackets

#### Entropy Rate and Excess Entropy

Figure 4 summarizes the results of entropy rate (A) and excess entropy (B) statistics for EEG microstate sequences in wake and sleep. While wakefulness and N1 sleep have a similar distribution of entropy rate (A) and excess entropy (B) values, entropy rate values decrease with deepening sleep (N2, N3) and excess entropy increases. Results for EEG data (dark grey) and first-order Markov surrogates (light grey) are similar. Two-way ANOVA analysis did not reveal any significant differences between EEG and surrogate data for entropy rate (p = 0.828) and excess entropy (p = 0.696). One-way ANOVA and post-hoc analysis over microstate sequence statistics revealed significant differences ($p_{FWE}<0.05$) between all vigilance states except W vs. N1. This significance pattern was found for both metrics, entropy rate and excess entropy.

#### Lempel–Ziv Complexity (LZC)

Lempel–Ziv complexity analysis of EEG microstate sequences in wake and sleep is shown in Fig. 4C. The numerical LZC values were re-scaled to bits/sample and are numerically very close to the entropy rate estimates in A. LZC values for Markov surrogates (light grey) were statistically not different from original data LZC values (two-way ANOVA, p = 0.881). When microstate sequence LZC values were analyzed separately, post-hoc comparisons showed significant differences ($p_{FWE}<0.05$) between all vigilance states except for the W-N1 comparison.

#### Hurst Exponents

Hurst exponent analysis of EEG microstate sequences in wake and sleep is illustrated in Fig. 4D. The difference between Hurst exponents from microstate sequences and surrogate sequences was significant (two-way ANOVA, $p=0.001$). One-way ANOVA of Hurst exponents for microstate sequences was significant ($p<0.05$), and pairwise post-hoc analysis revealed significant differences between all vigilance states except for the W-N2 contrast. The mean Hurst exponent in wakefulness was slightly larger than in N1 (difference: $-$0.034), while N2 and N3 showed larger Hurst exponents than wakefulness although the differences between the means were small (N2-W: 0.014, N3-W: 0.062).

**Fig. 5:**
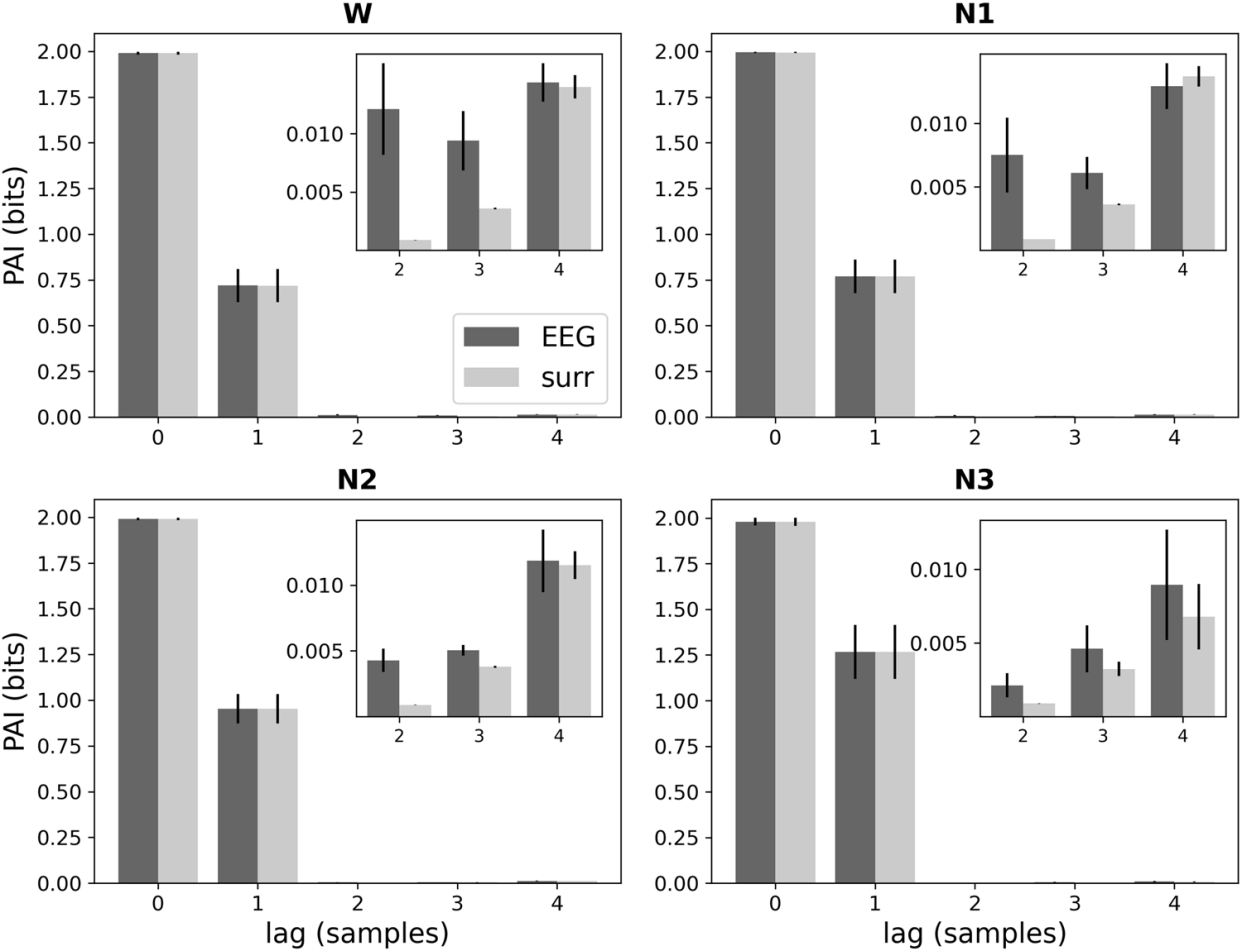
Partial autoinformation coefficients of EEG microstate sequences (‘EEG’, dark grey) and corresponding Markov surrogates (‘surr’, light grey) in wakefulness (W) and NREM sleep stages N1–N3. First-order Markov process properties are encoded in the coefficients for time lags 0 and 1. Insets focus on the higher-order coefficients (time lag 3–5 samples)

#### (Non-)Markov Structure of Microstate Sequences

To explain why complexity values for EEG data and first-order Markov surrogates were almost identical in Fig. 4, we analyzed the (non-)Markovian structure of EEG microstate sequences quantitatively. Figure 5 shows the partial autoinformation (PAI) coefficients of EEG microstate sequences in wakefulness and NREM sleep. As per construction of the surrogate data, PAI coefficients at time lags 0 and 1 were identical between data and surrogates. Higher-order coefficients (lags $\ge$ 2) had very small magnitude and are hardly visible in the main panels. Insets in Fig. 5 are re-scaled to make these non-Markovian components visible. Although differences between microstate and surrogate sequences exist in all states (W-N3), the relative contribution of higher-order coefficients to the overall sequence structure is very small.

#### Microstate Jump Sequences

**Fig. 6:**
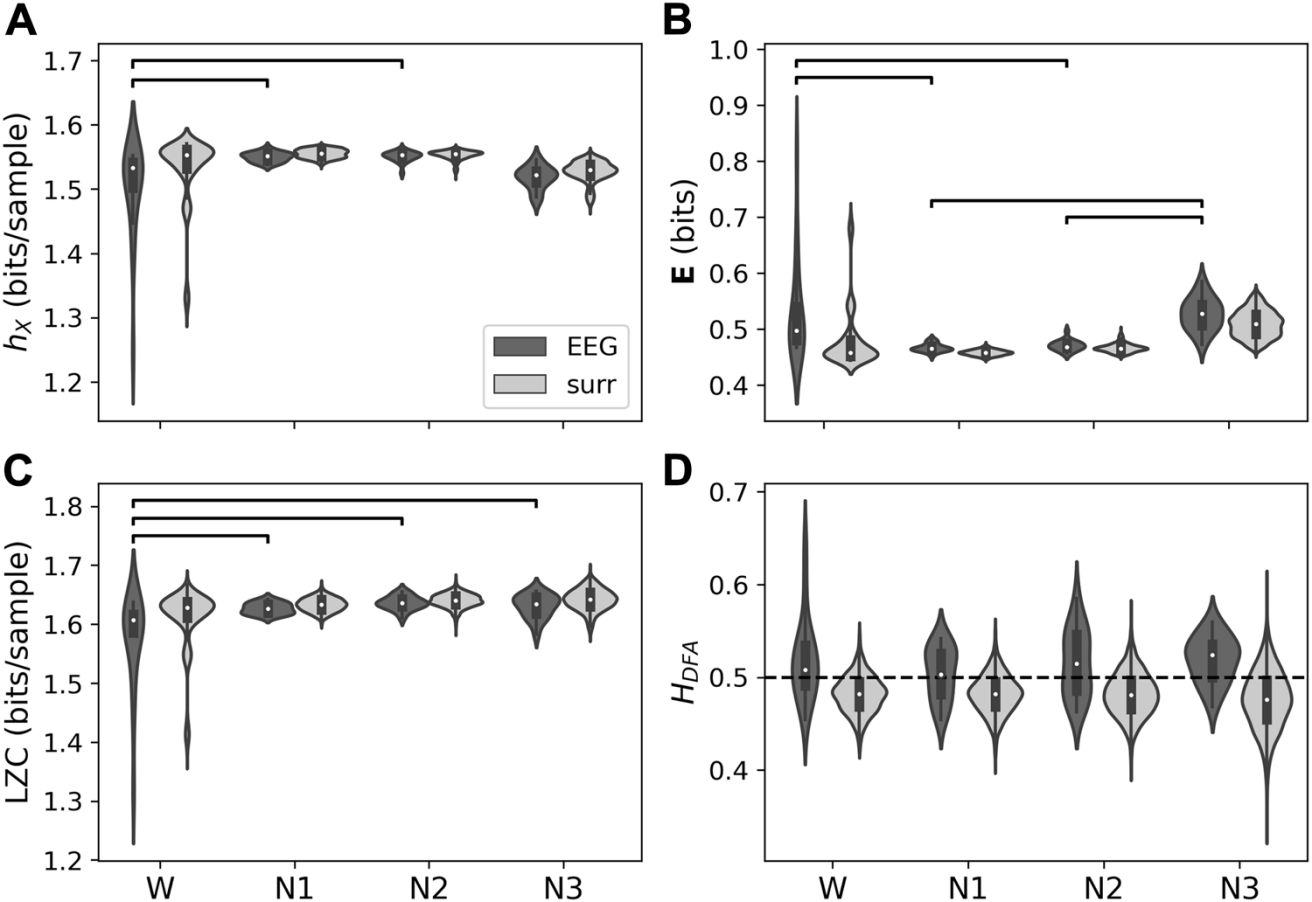
Complexity measures for EEG microstate jump sequences ($K=4$) in wakefulness and NREM sleep. Results for EEG microstate jump sequences are shown in dark grey (label ‘EEG’), Markov process surrogates in light grey (label ‘surr’). **A** Entropy rate ($h_X$), **B** excess entropy (${\textbf{E}}$), **C** Lempel–Ziv complexity (LZC), **D** Hurst exponents from DFA ($H_{\textrm{DFA}}$). Differences between microstate sequence results were tested with one-way ANOVA (significance level $\alpha =0.05$), and post-hoc Tukey tests. Significant pairwise differences ($p_{\textrm{FWE}}<0.05$) are indicated by brackets

Microstate jump sequences, i.e. sequences from which all duplicate labels have been removed, are shown in Fig. 6. Compared to full microstate sequences, jump sequences displayed more variable entropy rates and excess entropies in wakefulness (Fig. 6A, B). The entropy rate distribution was skewed towards lower values and excess entropy towards higher values. NREM sleep stage data had lower variability and we found statistically significant differences between W-N1 and W-N2. In contrast to the full sequences, mean entropy rates were slightly higher in sleep but the absolute differences were very small (N1-W: 0.0493 bits/sample, N2-W: 0.0499 bits/sample). Mean excess entropy values in light sleep were slightly lower than in wakefulness (W-N1: 0.0653 bits, W-N2: 0.0613 bits), but higher in N3 compared to light sleep (N3–N1: 0.0601 bits, N3–N2: 0.0561 bits). These differences should be interpreted with caution due to the highly skewed distributions in wakefulness, and due to biases of the estimation algorithms for short sequences, as will be explained below.

LZC values for microstate jump sequences also showed a high variability in wakefulness with a distribution skewed towards lower values. Significant differences were found between wakefulness and all three NREM sleep stages, with slightly larger mean values in sleep (mean differences N1-W: 0.053 bits/sample, N2-W: 0.0623 bits/sample, N3-W: 0.0568 bits/sample). In contrast to full microstate sequences, the numerical values of entropy rates and LZC values were different, with systematically larger LZC values.

#### Convergence of Entropy Rate and LZC

To investigate the difference between entropy rates and LZC values further, we analyzed the convergence rate of joint entropy ($h_X$) and LZC entropy rate estimators for Markov processes with a known entropy rate. To this end, we computed the cumulative transition probability matrix from all wakefulness microstate sequences and, based on this matrix, synthesized 50 first-order Markov surrogate sequences with length $n=10^5$ samples. We computed $h_X$ and LZC for sequences of different lengths, and Fig. S2 shows both metrics as a function of sequence length (mean and standard deviation). LZC converged towards the theoretical entropy rate ($h_{\textrm{theo}}=1.287$ bits/sample) from above, while $h_X$ approached $h_{\textrm{theo}}$ from below. The difference between $h_X$ and LZC for jump sequences in wakefulness was 0.073 bits/sample ($h_X$: 1.501 bits/sample, LZC: 1.574 bits/sample), and the lengths of jump sequences was in the range of 6923–10,287 samples. The observed difference between these two complexity metrics for microstate jump sequences ($h_X$ in Fig. 6A and LZC in Fig. 6C) matched the difference found for Markov processes of the same length and with known entropy rate (Fig. S2, grey area). The mean of $h_X$ for full microstate sequences in wakefulness was 1.245 bits/sample (Fig. 4A), and the mean LZC was 1.278 bits/sample (Fig. 4C). The convergence plot (Fig. S2) at n = 30,000 (lengths of full sequences) shows a difference of LZC-$h_X$=0.03 bits/sample, again showing close agreement with the difference observed for EEG microstate sequences.

The effects of deleting repeated microstate labels on Hurst exponents is quantified in Fig. 6D. Hurst exponents were estimated for microstate jump sequences and their first-order Markov surrogates. Both microstate sequences and Markov surrogates had Hurst exponents distributed around $H=0.5$, the expected value for processes without long-range correlations. Pairwise differences were not statistically significant. Thus, removal of duplicate symbols erased long-range correlations observed in full microstate sequences (Fig. 4D).

## Discussion

The main results of this report can be summarized as follows:

- Kolmogorov complexity and statistical complexity are two fundamentally different concepts of complexity and the existing EEG microstate studies have focused on the former concept (randomness).
- We evaluated excess entropy for microstate sequence analysis and found that it (i) correctly identifies the critical point in the Potts model and (ii) increases with deepening NREM sleep stage in EEG microstate sequences.
- Re-scaled Lempel–Ziv complexity and entropy rate are equivalent metrics of microstate complexity if the LZ-76 implementation is used and if sequences have sufficient length. Both metrics represent the Kolmogorov complexity concept.
- Entropy-related measures including LZC are determined by first-order dependencies of EEG microstate sequences, whereas Hurst exponents integrate information from longer time scales. Limitations of Hurst exponent analysis were observed for the low-temperature Potts model.
- Microstate jump sequences display more randomness than full sequences and do not preserve long-range correlations.

### The Potts Model and EEG Microstate Sequences

We decided to evaluate the selected complexity metrics on Potts model data before studying EEG data. The Potts model is a widely studied system in statistical physics with a discrete state space and a well-defined phase transition (Wu 1982). The rationale for this approach was that it allowed us to gauge the behaviour of complexity metrics at defined system states whereas the ground truth complexity of a brain state measured in an experiment is unknown. We used the Potts model variants with $Q=4$ and $Q=5$ discrete states, respectively, for two reasons. First, most EEG microstate analyses use either four or five microstate classes (Michel and Koenig 2018). The second reason was that the 2D Potts model for $Q=4$ undergoes a second-order phase transition and a first-order phase transition for $Q=5$. Although there is evidence that resting-state brain activity shares phase-transition like features with physical models (Chialvo 2010; Tagliazucchi et al. 2012), the type of phase transition occurring in the brain is unknown in general. Overall, all tested metrics showed the same characteristics for $Q=4,5$, except for the anticipated differences in absolute entropy values which depend on *Q*. We therefore conclude that the tested metrics do not depend on the type of phase transition.

### Entropy Rate and Excess Entropy

We have found entropy rate and excess entropy to reflect the concepts of Kolmogorov complexity and statistical complexity, respectively. Their difference can be expressed in terms of forecasting (Grassberger 1986). Kolmogorov complexity describes how hard it is to predict a signal correctly, and statistical complexity measures how hard it is to find the best predictor. The difference between these two approaches becomes evident when applied to purely random patterns. They are impossible to predict precisely (high entropy rate and LZC), but the best predictor can be found easily. A microstate sequence without any temporal order, for example, is hard to predict but the best estimator is a simple bet on the relative probabilities of occurrence of each microstate, e.g. 1/4 each if uniformly distributed. Statistically complex patterns, however, can be easier to predict, but the best predictor can be impossible to find as it might need to capture the full non-linear dynamics of the underlying system.

One of the aims of this article was to explore excess entropy as a potentially useful addition to EEG microstate analysis. This raises the question whether excess entropy can contribute anything to the information already gained from other metrics. The answer is that excess entropy reflects the concept of statistical complexity which is not captured by either entropy rate or Lempel–Ziv complexity, and that its value cannot be predicted from these other metrics. The latter point is illustrated by the Potts model results which show that excess entropy and entropy rate can covary either positively or negatively. For sub-critical temperatures ($T/T_c<1$ in Fig. 3), both metrics increase with temperature. At low temperatures, future states are not difficult to predict as there are few state switches and conditioning on the past hardly improves the prediction. Approaching the critical temperature, future states become increasingly difficult to predict (higher entropy rate) but at the same time knowledge of the past significantly improves the prediction (higher excess entropy). Improved predictability is explained by expanding autocorrelations near the phase transition which can be exploited to make predictions less uncertain. In information-theoretic terms, we find more shared information between the past and the future states of the sequence near the critical point. Above the critical temperature, randomness (entropy rate) increases further but dependencies on past states fade as sequences approach the uncorrelated noise level.

Another metric called sample entropy, a measure related to entropy rate, was evaluated in Murphy et al. (2020) where an almost random pattern of microstates in psychosis patients was observed. It must be noted, however, that (Murphy et al. 2020) analyzed microstate jump sequences. Our analyses show that these sequences, due to the removal of duplicate labels and microstate duration statistics, display higher levels of randomness and cannot capture long-range dependent sequence features, as further discussed below.

### Lempel–Ziv Complexity

A collection of data compression algorithms developed in the 1970s are associated with the names of Lempel and Ziv, notably the LZ-76, LZ-77, and LZ-78 algorithms (Lempel and Ziv 1976, 1977; Ziv and Lempel 1978). Despite their similar names, only the LZ-76 algorithm has a close relationship to theoretical quantities like the entropy rate, whereas later versions were optimized for practical data compression tasks.

The main results were derived in Ziv (1978) where it was shown that LZC (LZ-76 algorithm) provided an exact estimate of entropy rate if the underlying stochastic process is stationary. Stationarity cannot be tacitly assumed for EEG signals in general (von Wegner et al. 2017), but the theoretical link provided a starting point for the analyses in this article. A similar hypothesis was put to test for neural spike train data in Amigó et al. (2004) where the earlier LZ-76 algorithm proved to be numerically equivalent to the entropy rate of binarized spike trains, whereas LZ-78 contained a marked bias relative to the true entropy rate.

In EEG microstate research, Lempel–Ziv complexity (LZC) was first used by Tait et al. (2020), and subsequently by Artoni et al. (2022). The former article uses the authors’ LZ-76 implementation (plus-microstate toolbox) whereas the latter used the dictionary-based LZMA2 algorithm from the popular 7zip software (7zip). In analogy to (Amigó et al. 2004), we found that normalized LZC output from the LZ-76 algorithm provided excellent numerical agreement with the entropy rates computed from joint probability distributions as evident from comparison between the top and center panels of Fig. 3.

On the other hand, we also observed that this numerical agreement relies on sample size and discrepancies are more pronounced for shorter sequences as explained by the convergence behaviour of the two metrics.

Entropy rate and LZC have so far been discussed separately in the microstate literature (von Wegner et al. 2018a; Jia et al. 2021; Tait et al. 2020; Artoni et al. 2022; Wiemers et al. 2023). The equivalence of both metrics means that LZC values as those given in Tait et al. (2020) and entropy values we published in different contexts (von Wegner and Laufs 2018; Jia et al. 2021; Wiemers et al. 2023) can be re-scaled and compared directly. Unfortunately, this is not possible for LZC values computed by more recent versions of the LZ algorithm, for example the complexity values given in Artoni et al. (2022). Complexity metrics computed from the LZ-77 and LZ-78 algorithms are likely to yield similarly shaped curves to entropy rate and LZ-76 plots in Fig. 3, but the LZ-76 implementation has the advantage of being a direct numerical estimator of the theoretically meaningful entropy rate.

### Hurst Exponents

Fractal dimension and Hurst exponent analysis are based on a different theoretical framework than entropy-based methods, and they can be found as complexity measures in functional MRI studies (Bullmore et al. 2009; Tagliazucchi et al. 2013; Dong et al. 2018), EEG studies (Linkenkaer-Hansen et al. 2001; Raghavendra et al. 2009; Sabeti et al. 2009; Holloway et al. 2014), but also outside brain research (Raubitzek and Neubauer 2021).

Applied to Potts model data, Hurst exponents represent statistical complexity in a similar way to excess entropy. In contrast to the entropy-related metrics including LZC, Hurst exponents for Markov surrogates had markedly lower Hurst exponents. This could be observed for Potts model data (Fig. 3) as well as for EEG microstate sequences (Fig. 4). This demonstrates that the inclusion of larger time scales up to 10 s in the DFA parameters identified time series properties that were not visible to the entropy-based methods. This observation may appear obvious because entropy rate/excess entropy calculations were based on a finite history length of only $k=6$ samples. Lempel–Ziv analysis, however, does not contain an explicit time window and progressively scans the whole time series and keeps track of previously seen patterns. Yet, LZC values were identical to entropy rate values which shows that there was no additional information on larger time scales through the Lempel–Ziv lens. In contrast to Lempel–Ziv analysis, DFA does not search for actual repetitions of exact patterns but accepts anything that contributes variance at a given time scale. Although DFA quantifies self-similarity in signals that exhibit this property, it is not a generic detector of self-similarity in the strict sense. From the Hurst exponents observed in Figs. 3 and 4 that Potts model data and EEG microstate sequences in deeper sleep stages display slow fluctuations that are not explained by exact repetitions of signal segments.

Potts model data analysis also demonstrated an important pitfall of DFA and Hurst exponent analysis. The large variability of Hurst exponents observed for the cold Potts model in Fig. 3 is caused by time series with a very small number of state changes. These cause isolated step-like jumps when the signal is transformed into a random walk according to the technique proposed for microstate sequences (Van de Ville et al. 2010). Jumps in turn lead to large fluctuations at long time time scales and empirically result in large Hurst exponents $H>1.5$ although the signal is constant most of the time. When zero state changes occur, however, the expected value of $H=0.5$ was obtained. This leads to the undesired result that near-constant time series that differ only in a small fraction of values result in massively different Hurst exponents. Again, this is related to the DFA algorithm being a variance analyzer. Non-stationarities such as steps in the signal add variance over a range of time scales that could be misinterpreted as a mono-fractal signature from the value of the Hurst exponent alone. It should also be noted that we have not encountered near-constant time courses over hundreds or thousands of samples for EEG microstate sequences, and we believe that the problem discussed for the cold Potts model is not an issue for EEG microstate analysis if the sequence length is at least a few hundred samples.

We conclude that the Hurst exponent of a microstate sequence can (i) be interpreted as a marker of statistical rather than algorithmic complexity, and (ii) provides a perspective on short and long time scales whereas the entropy-related measures we evaluated reflect short-range dependencies.

### Microstate Sequence Complexity in Wake and Sleep

In this study, we have applied excess entropy, LZC, and DFA as novel methods to characterize the complexity of microstate sequences from wakefulness and NREM sleep. Entropy rate analysis was presented in Wiemers et al. (2023) but is shown again here with additional Markov surrogate comparisons. Our main findings are that deepening sleep stages were accompanied by decreasing entropy rate and LZC, increased excess entropy, and increasing Hurst exponents.

Microstate jump sequences did not show long-range correlations and overall less pronounced differences between sleep stages.

The reduced entropy rate and LZC values are probably related to the progressive slowing of EEG rhythms in sleep. Longer microstate duration (Wiemers et al. 2023) improves the predictability of microstate sequences in sleep stages N2 and N3 and reduce their apparent randomness. This result is similar to the findings of Tait et al. who found EEG slowing and reduced LZC values in Alzheimer disease patients although they corrected their microstate analysis for background EEG slowing effects (Tait et al. 2020; Dauwels et al. 2011).

From the discussion of the Potts model it is clear that the observation of entropy rate changes cannot predict changes in excess entropy. We found increased excess entropy values in N2 and N3, which corresponds to the Potts model situation above the critical temperature. Within this analogy, the transition to deeper sleep stages corresponds to a shift from higher temperatures towards the critical point. This is intuitively correct as deep sleep is accompanied by EEG signals with longer, delta rhythm-related autocorrelations which translate into increased shared information between past and future microstates. This explanation is further substantiated by our recent finding that microstate oscillations in the delta frequency range occur in N3 (Wiemers et al. 2023), and by the larger Hurst exponents in N2 and N3 (Fig. 4D). The straightforward conclusion from this analogy would be to classify deeper sleep stages as being closer to a critical system state. This, however, at least partially contradicts earlier studies using EEG and other imaging modalities.

Weiss et al. found larger Hurst exponents in deep sleep stages using the DFA-related R/S statistic on raw EEG data from humans (Weiss et al. 2009). In rats, local field potential measurements revealed a similar trend, i.e. a lower complexity (LZC) in NREM vs. wakefulness (Abásolo et al. 2014). Another measure called Omega complexity, based on the eigenvalue spectrum of the spatial principal component analysis of an EEG data set, is known to decrease in deeper NREM sleep stages as well (Wackermann 1996). Not all analyses point in the same direction however. Kantelhardt et al. performed an extensive analysis of Hurst exponents across sleep stages and frequency bands and found different trends for different frequency bands. In the alpha band, the main contributor to microstate topographies (Milz et al. 2017), the authors found lower Hurst exponents in deeper sleep, contradicting our results for microstate sequences. The opposite trend, however, was reported for other frequency bands (Kantelhardt et al. 2015). Different outcomes might also be due to the pre-processing of the input signals. Frequency band analyses often use the power (envelope) of narrow frequency band oscillations and not the oscillatory signal itself (Linkenkaer-Hansen et al. 2001; Kantelhardt et al. 2015), whereas microstate analysis starts with broad-band EEG oscillations and does not use the envelope signal. When a different recording modality is used, the results can change once again. In the past we have observed smaller Hurst exponents during sleep in regional BOLD (blood oxygen level dependent) signals from functional MRI data, and interpreted those as a departure from criticality, concluding that wakefulness was the state closest to criticality (Tagliazucchi et al. 2013). The methodological differences and the nature of the physiological signal used might explain why electrophysiological and imaging data are unable to give a unique answer to the question how close the brain is to a critical state in a defined condition.

Another difference between the Potts model and EEG data is that the Potts model is controlled by a single parameter (temperature) that, when varied continuously, controls the extent of autocorrelations in the data. Sleep stage transitions, on the other hand, involve a switching between different system states in which different frequency generating circuits are active, namely alpha generators in wakefulness and delta/slow wave generators in N3. Furthermore, sleep contains isolated events never observed in wakefulness (vertex sharp waves, sleep spindles, K-complexes) (Adamantidis et al. 2019). From electrophysiological studies we know that different neuron populations partake in these patterns and that their voltage responses are fundamentally different, e.g., spiking vs. bursting (Llinás and Steriade 2006). Similar heterogeneity is not modelled by the Potts system and demonstrates limitations of statistical physics models of brain activity. A shift from alpha to delta frequency band activity, with corresponding repercussions on microstate patterns as observed in Wiemers et al. (2023) alone can probably explain a reduced entropy rate, increased excess entropy and larger Hurst exponents due to larger variance contributions at long time scales. The interpretations with regard to Kolmogorov and statistical complexity are unequivocal, yet, in the absence of an experimentally controllable parameter we would hesitate to strictly interpret this as the same system moving closer to a phase transition. This might be possible in modeling studies though, which indicate that phase transition-like mechanisms might indeed play a role in wake-sleep and anesthesia-induced transitions (Steyn-Ross et al. 2004, 2005). Further discussions about the complexity of brain activity during sleep can be found in Olbrich et al. (2011) and references therein.

Another pre-processing strategy for microstate sequences is the deletion of duplicate symbols, a process that only retains the jumps between non-identical states. This approach is rooted in Markov chain theory where the result is called the jump process or jump chain (Gillespie 1992). In the microstate context, this has been termed the transitioning sequence (Tait et al. 2020), compressed sequence (Murphy et al. 2020), and no-permanence sequence (Artoni et al. 2022, 2023). We prefer the well-established term jump process to emphasize the link to Markov chain theory and the large body of literature that can be found under that name. The results for jump processes derived from EEG microstate sequence in wakefulness and sleep were markedly different from the full sequences. Entropy rate, excess entropy and LZC had a large variability in wakefulness and the skewed distributions observed in Fig. 6 are likely responsible for the significant differences between wakefulness and sleep stages. The absolute differences were minute compared to full microstate sequences and we would interpret them with caution, given the skewed wakefulness distributions. Another reason to treat these results with reserve is that jump sequences become shorter with deepening sleep stage (W: 6923–10,286, N1: 6129-,10,264, N2: 4903–7474, N3: 2248–6513 samples). This effect is due to longer microstate duration in sleep (Wiemers et al. 2023). As shown in Fig. S2, entropy rate estimators have a significant bias for shorter sequences and comparisons between sleep stages are likely to yield false positive results due to estimator biases. This was not a problem for full sequences which all had 30,000 samples. Nevertheless, the overall differences between full and jump sequences included higher entropy rates/LZC values and lower excess entropies for jump sequences. This effect can be readily explained. Removal of duplicate microstate labels from a sequence increases its apparent randomness. The transition probability matrices of full sequences have their largest entries along the diagonal, i.e., A-to-A transitions are far more likely than any other A-to-X transition, and similarly for other states. Concrete values were given in von Wegner et al. (2017), for instance. Thus, any occurrence of label A in a full sequence increases the predictability over the next few time steps, and this reduces the entropy rate/LZC values compared to the jump sequences in which repetitions never occur.

Another feature of jump sequences is that the information about microstate lifetimes is lost. Yet, non-exponential lifetime distributions are a well-understood mechanism underlying long-range correlations (Clegg and Dodson 2005). It is therefore not surprising to find Hurst exponents indistinguishable from 0.5 for all jump sequences and their Markov surrogates (Fig. 6D). Analogous results were already reported in the original study investigating LRD in random-walk embedded microstate sequences (Van de Ville et al. 2010). These results demonstrate that researchers interested in long-range phenomena must focus on full microstate sequences as these features are removed when passing to the jump sequence representation.

### Practical Considerations and Limitations

The results presented above can be rephrased as a list of practical suggestions and considerations for complexity analyses of EEG microstate sequences.

(i) The LZ-76 algorithm is fast and computationally less expensive than joint entropy estimations and is therefore an appealing practical alternative to computing entropy rates. The approach has potential applications for statistical comparisons of EEG microstate sequences with non-trivial surrogate data. By trivial, we refer to surrogates devoid of temporal correlations, as those obtained by simple shuffling of microstate sequences. The null hypothesis of zero autocorrelations is tested with Lehmann’s syntax analysis (Lehmann et al. 2005). A minimum layer of complexity is added by modeling first-order autocorrelations between states at time *t* and $t+1$. These are captured by first-order Markov surrogates as used in (von Wegner et al. 2016, 2017). The theoretically plausible and now empirically confirmed (near-)equivalence between entropy rates and LZC values for microstate sequences allows the prediction of LZ complexity under a Markov null hypothesis. Practically, the analytical entropy rate under a Markov null hypothesis can be calculated from the empirical microstate transition matrix, thereby avoiding the cost of synthesizing large numbers of surrogate sequences.
(ii) A practical argument in favor of the LZ-76 algorithm is that there are no free parameters that need to be set by the researcher and that will affect the numerical complexity values returned by the algorithm. The free parameters of the LZMA2 algorithm (compression level, dictionary size, number of fast bytes, filter and match-finder options, Artoni et al. 2022) are useful to achieve a maximum compression level for different data types but they have no evident theoretical meaning, and the quantitative relationship with the actual entropy rate needs to be established. Nevertheless, LZMA is a practical method of measuring microstate sequence compressibility.
(iii) The joint entropy approach to entropy rate estimation has the advantages that (a) it returns excess entropy ’for free’ from the same linear fit that is used to obtain entropy rate, and (b) the joint entropy values for microstate words of a given length might be of interest for certain research questions. For instance, a recent article on microstate sequence syntax analysis is based on word entropies (Artoni et al. 2023).
(iv) All approaches are limited by finite sample size effects. In particular, the equivalence between entropy rate and LZC becomes numerically imprecise for short microstate sequences.
(v) Hurst exponent analysis can become unreliable when very few state changes occur. We have encountered this case for model data only.

### Conclusion

The concepts of Kolmogorov and statistical complexity are useful to structure future discussions about EEG microstate sequence complexity. The results of this study, namely the evaluation of excess entropy, the demonstrated equivalence between entropy rate and LZC, and the systematic comparison with Hurst exponent analysis are meant to add to the theoretical and practical framework of this research area.

### Supplementary Information

Below is the link to the electronic supplementary material.

Supplementary material 1 (PDF 111 kb)

Supplementary material 2 (PNG 421 kb)

Supplementary material 3 (PNG 141 kb)
