# Supplementary material for: Complexity Measures for EEG Microstate Sequences: Concepts and Algorithms
Source: Brain Topogr. 2023 Sep 26;37(2):296–311. doi: 10.1007/s10548-023-01006-2 (PMC10884068; doi:10.1007/s10548-023-01006-2)
Supplement: Supplementary file 1 — Supplementary material 1 (PDF 111 kb) [file 10548_2023_1006_MOESM1_ESM.pdf]

Supplemental Data

Potts model ($Q = 5$)

In analogy to Figure 3, we here present the results for the five-state Potts model ($Q = 5$) in Figure S1. The model has a first-order phase transition whereas the $Q = 4$ model undergoes a second-order phase transition. Both models show highly similar results with regards to the investigated complexity parameters. The absolute entropy rate and excess entropy rate values depend on Q and are therefore different from $Q = 4$. The maximum entropy rate for $Q = 5$ is 2.32 bits/sample.

Entropy rate convergence

Entropy rate estimates depend on sample size, i.e. the lengths of the analyzed sequences. The convergence of the two estimators, entropy rate (h_X) and LZC, is shown for different sequence lengths in Figure S2. We first computed the compound transition probability matrix from all EEG microstate sequences in wakefulness. This transition matrix was used to generate a set of $n = 50$ first-order Markov sequences with 10^5 samples each. For Markov surrogates, the theoretical entropy rate can be calculated analytically from the transition matrix T_{ij} and the equilibrium state distribution π_i as $-\sum_{ij} \pi_i T_{ij} \log(T_{ij})$. We then computed h_X and LZC for Markov sequences truncated to different sample sizes, and the results are shown in Figure S2. It is observed that both estimators approach the theoretical entropy rate of 1.287 bits/sample asymptotically, however, h_X approaches the theoretical value from below whereas LZC approaches from above. The range of actual EEG microstate jump sequence lengths in wakefulness is indicated by the grey area. Across that range, the difference between h_X and LZC is similar to their difference for microstate jump sequences in wakefulness as observed in Figure 6A and Figure 6C. We therefore conclude that these differences in Figure 6 are due to sample size and are expected to disappear for longer sequences.
