# Supplementary figures and images for: Complexity Measures for EEG Microstate Sequences: Concepts and Algorithms
Source: Brain Topogr. 2023 Sep 26;37(2):296–311. doi: 10.1007/s10548-023-01006-2 (PMC10884068; doi:10.1007/s10548-023-01006-2)

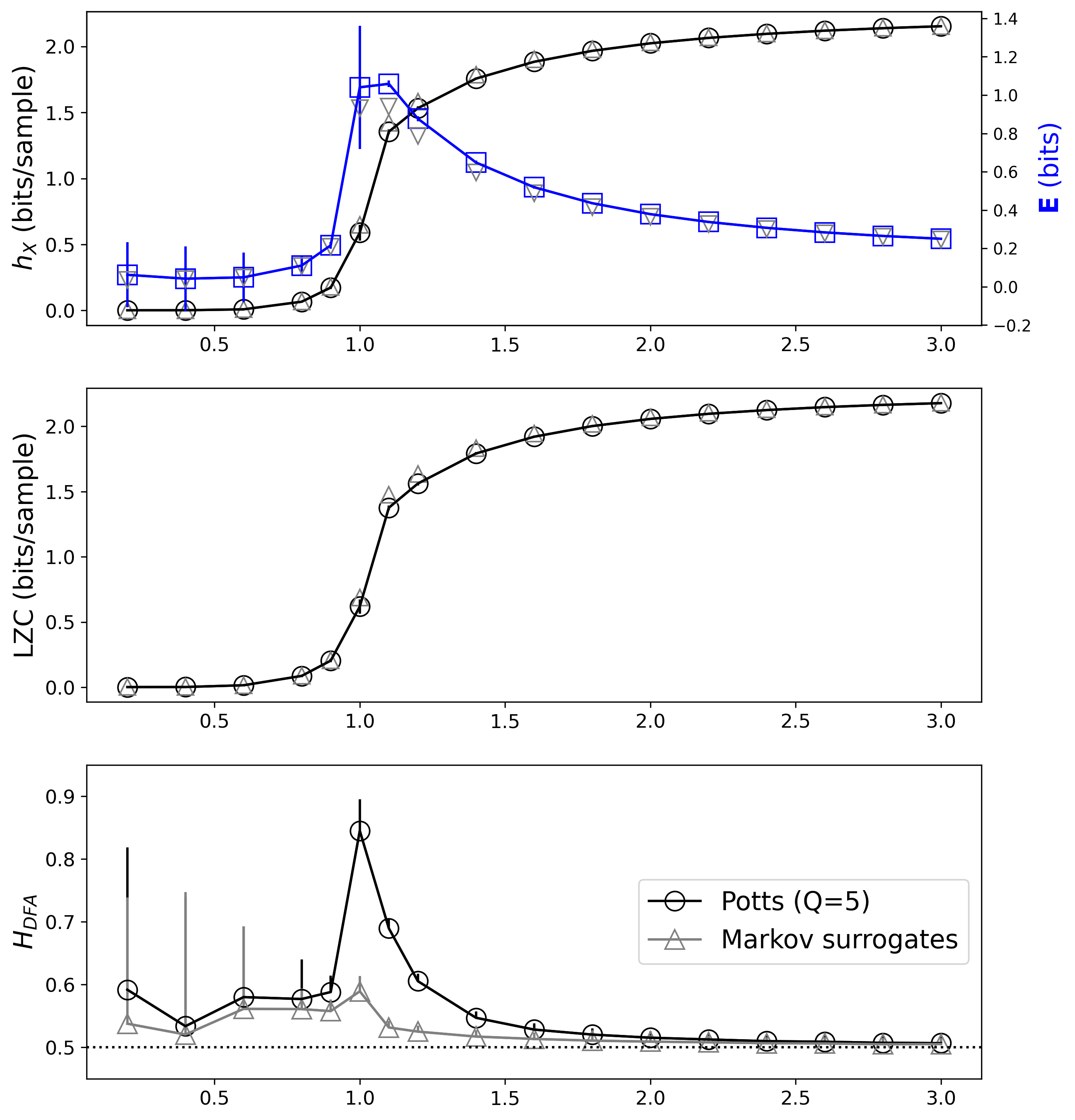

Supplement: Supplementary file 2 — Supplementary material 2 (PNG 421 kb) [file 10548_2023_1006_MOESM2_ESM.png]

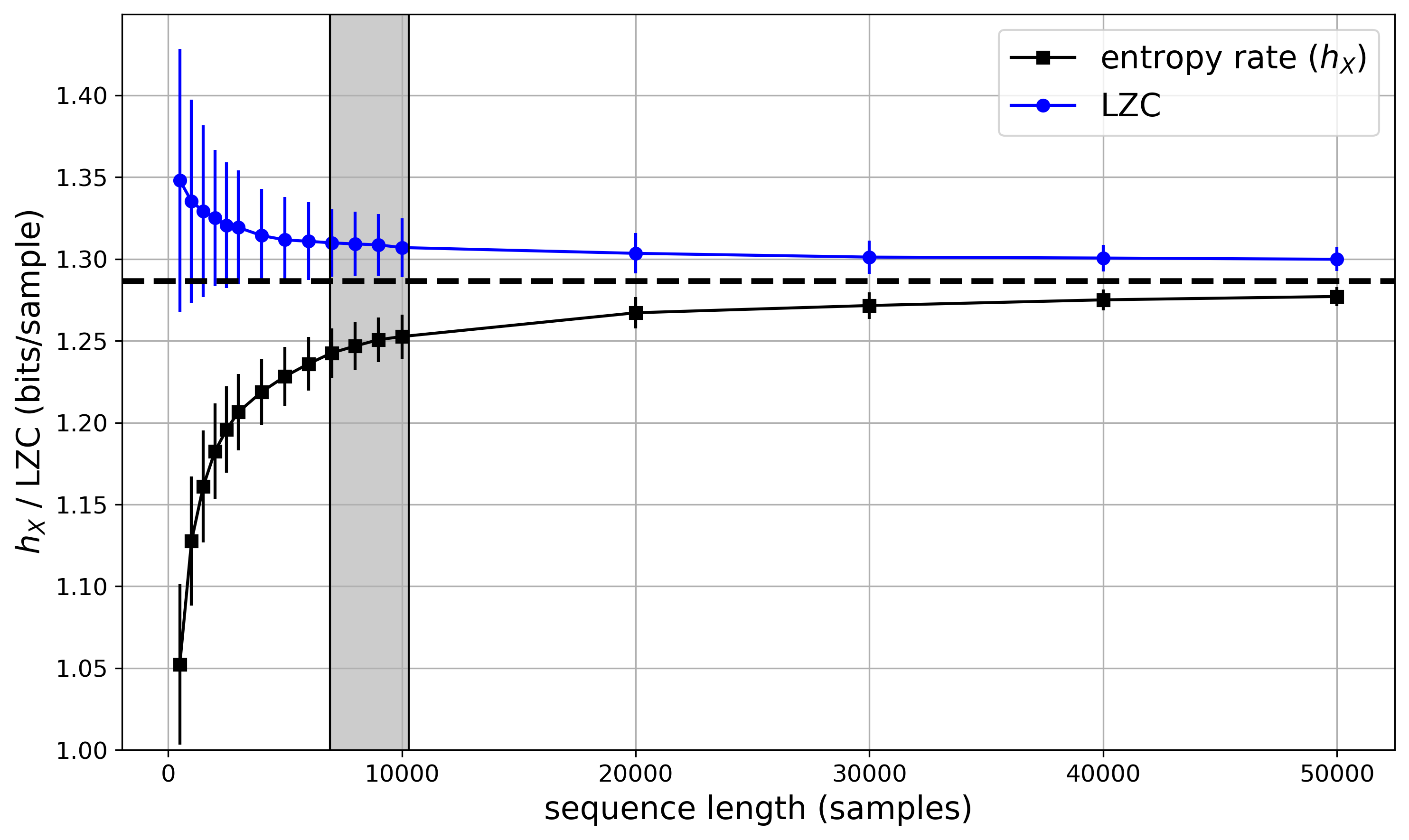

Supplement: Supplementary file 3 — Supplementary material 3 (PNG 141 kb) [file 10548_2023_1006_MOESM3_ESM.png]
